# Detection of *Mycosphaerella graminicola* in Wheat Leaves by a Microsatellite Dinucleotide Specific-Primer

**DOI:** 10.3390/ijms12010682

**Published:** 2011-01-19

**Authors:** Kamel Abd-Elsalam, Ali H. Bahkali, Mohamed Moslem, Pierre J. G. M. De Wit, Joseph-Alexander Verreet

**Affiliations:** 1 Institute of Phytopathology, Christian-Albrechts-University Kiel, Hermann-Rodewald-Str. 9, D-24118, Kiel, Germany; E-Mail: javerreet@phytomed.uni-kiel.de; 2 Botany and Microbiology Department, College of Science, King Saud University, P.O. Box: 2455, Riyadh 1145, Saudi Arabia; E-Mails: abhakali@ksu.edu.sa (A.H.B.); mbmoslem@ksu.edu.sa (M.M.); 3 Plant Pathology Research Institute, Agricultural Research Centre, Giza, Egypt; 4 Laboratory of Phytopathology, Wageningen University, Droevendaalsesteeg 1, NL-6708 PB Wageningen, The Netherlands; E-Mail: pierre.dewit@wur.nl

**Keywords:** *Septoria tritici* blotch, microsatellite, wheat, Dothidiomycete, molecular diagnostics

## Abstract

Early detection of infection is very important for efficient management of *Mycosphaerella graminicola* leaf blotch. To monitor and quantify the occurrence of this fungus during the growing season, a diagnostic method based on real-time PCR was developed. Standard and real-time PCR assays were developed using SYBR Green chemistry to quantify *M. graminicola in vitro* or in wheat samples. Microsatellite dinucleotide specific-primers were designed based on microsatellite repeats of sequences present in the genome of *M. graminicola.* Specificity was checked by analyzing DNA of 55 *M. graminicola* isolates obtained from different geographical origins. The method appears to be highly specific for detecting *M. graminicola*; no fluorescent signals were observed from 14 other closely related taxa. Primer (CT) 7 G amplified a specific amplicon of 570 bp from all *M. graminicola* isolates. The primers did not amplify DNA extracted from 14 other fungal species. The approximate melting temperature (Tm) of the (CT) 7 G primer was 84.2 °C. The detection limit of the real-time PCR assay with the primer sets (CT) 7 G is 10 fg/25 μL, as compared to 10 pg/25 μL using conventional PCR technology. From symptomless leaves, a PCR fragment could be generated two days after inoculation. Both conventional and real-time PCR could successfully detect the fungus from artificially inoculated wheat leaves. However, real-time PCR appeared much more sensitive than conventional PCR. The developed quantitative real-time PCR method proved to be rapid, sensitive, specific, cost-effective and reliable for the identification and quantification of *M. graminicola* in wheat.

## 1. Introduction

*Septoria tritici* blotch (STB) of wheat (*Triticum aestivum* L.), caused by the fungal pathogen *Mycosphaerella graminicola* (anamorph: *Septoria tritici*), occurs in all wheat-growing areas world-wide, with an increasing economic impact over the last decades [1]. The causal agent, *S. tritici*, was first described by Desmazières [2]. Sanderson identified the ascomycete *M. graminicola* as the sexual stage (teleomorph) of *S. tritici* [3,4]. The origin of *M. graminicola* is most likely the Middle East [5]. *M. graminicola* is developing rapidly as a model for fungi in the order Dothideales [6,7]. Presently the fungus represents a major economic concern for global wheat production [8].

Polymerase chain reaction (PCR) methods that record fluorescence in real time when samples pass photo detection diodes have been described. This can be achieved by using double-stranded DNA-specific dyes, such as SYBR green (SG). The sensitivity of this method is similar or better than other PCR methods [2]. Recently, PCR-based real-time quantitative assays have been developed for the detection of a variety of plant pathogens [9–11], enabling high throughput diagnostics of plant pathogen infection in a relatively short time.

Specific PCR tests have been developed for identification of *M. graminicola* in wheat [12–15]. A PCR system in a fluorescent amplification-based specific hybridization (FLASH) format was developed for the detection and identification of *M. graminicola* [16]. Microsatellites and repeat sequence polymorphisms have been studied previously using real-time PCR chemistries such as hybridization probes [17,18]. Microsatellites have been used in several epidemiological studies of phytopathogenic fungi [19,20].

Our reasoning for testing microsatellites as a diagnostic tool for *M. graminicola* is based on the unique characteristics of this class of markers. Microsatellites are short tandem repeats of a simple nucleotide sequence, inherited in a Mendelian fashion, and are evenly distributed in the genome. Although we could not test every potential non-target, our assumption is that microsatellites are usually species-specific [21,22]. Also, presymptomatic and accurate diagnosis of viable pathogen structures in infected wheat plants is desirable for determining latent periods of epidemics and for timely treatments with fungicides.

Conventional methods to detect and identify fungal pathogens in crop plants are time consuming, laborious, and require skilled taxonomical expertise [23,24]. Therefore, a PCR-based assay was developed with *M. graminicola*-specific primers designed on the repeat motif. The objective of this study was to develop a quantitative PCR (qPCR) assay based on the detection of the SYBR Green dye for the quantitative assessment of *M. graminicola* in wheat tissues.

## 2. Experimental Section

### 2.1. Fungal Isolates and Growth Conditions

Fifty-five isolates of *M. graminicola* and other fungal species used in the current study are listed in Table 1. Isolates used for DNA isolation were grown on yeast glucose broth (YG; 1% yeast extract, 3% glucose) at 18 °C by shaking for five days on an orbital shaker at a speed of 120 rpm. Spores were collected from YG by centrifugation and washed three times with sterile water and subsequently once with 0.6 M MgSO4 (pH 5.8). For storage for three-to-six months, the *M. graminicola* isolates were cultured on Malt Yeast Agar (MYA) plates then stored at 4 °C or −80 °C in PD broth medium supplemented with 10% glycerol [14].

### 2.2. Artificial Inoculation of Wheat Plants and DNA Isolation

Seeds of cultivar Ritmo (a highly susceptible wheat cultivar) were germinated on filter paper in the dark at 25 °C. After 24 h, the seeds were placed at 5 °C for 48 h followed by incubation at 25 °C for 24 h. The plants were then transferred to a growth chamber at 19 °C, with a day/night regime of 16 h/8 h. Wheat seedlings were inoculated by placing 10 μL of *M. graminicola* pycnidiospore suspension (containing 10^6^ spores) on each emerged leaf (leaf 2). Control plants were treated with distilled water. The inoculated plants were kept in a mist chamber for 72 h and then returned to the growth chamber at 21 °C (day)/16 °C (night) temperatures with a day/night regime of 16 h/8 h. Samples for PCR analysis were collected at 0, 2, 6, 8, 10, 12, 14, 16, 18 and 20 days after inoculation. DNA was extracted from wheat leaves according to procedures described by Guo *et al.* [15].

### 2.3. DNA Isolation

A modification of the traditional sodium dodecyl sulfate (SDS) extraction procedure was adopted [1]. Fungal mats (100 mg) were harvested and homogenized in 400 μL of sterile salt homogenizing buffer (200 mM Tris-HCl, pH 8.5, 250 mM NaCl, 25 mM EDTA, 0.5% SDS). Then, 6 μL of RNase A (final concentration 20 mg mL^−1^) was added and mixed. The samples were incubated at 65 °C for 10 min, after which 130 μL of 3 M sodium acetate, pH 5.2, was added to each sample. Samples were mixed in a Vortex for 30 s at maximum speed, and incubated at −20 °C for 10 min. The lysate was centrifuged at 13,000 rpm at 4 °C for 15 min. The supernatant was transferred to fresh tubes. An equal volume of isopropanol was added to each sample, mixed, and samples were incubated at −20 °C for 10 min. Samples were then centrifuged for 20 min at 4 °C, at 6,000 rpm. DNA pellets were washed twice with 700 μL of washing solution (100% and 70% ethanol, respectively). The DNA pellets were subsequently air-dried in an oven at 40 °C for at least 10 min. The resultant DNA pellet was then resuspended in 100 μL of 1 × TE (10 mM Tris-HCl, 1 mM EDTA) buffer, pH 8.0.

### 2.4. Microsatellite Selection and Design of PCR Primers

Microsatellites of varying length and type (di or trinucleotide) were selected from those identified by Karaoglu *et al.* [25], which are listed at http://www.mmrl.med.usyd.edu.au/an_contig.html. Microsatellite dinucleotide specific-primers were designed [(CTC TCT CTC TCT CT) G] based on microsatellite repeat sequence selected from the finished genome sequence of isolate IPO323 of *M. graminicola* (http://genome.jgi-psf.org/Mycgr3/Mycgr3.home.html).

### 2.5. Microsatellite-Primed PCR (MSP-PCR)

PCR mixtures contained 2 mM deoxynucleoside triphosphates, 10 pmol concentrations of primer; 0.2 U of *Taq* polymerase (Biolab, England), 1 × buffer (Promega, Mannheim, Germany), 1.5 mM MgCl_2_ and 50 ng of template DNA in a total volume of 25 μL. The reactions were carried out in a PTC-200 Thermocycler (MJ Research, Waltham, USA) as follows: 1 cycle of 2 min at 94 °C followed by 40 cycles of denaturation at 94 °C for 1 min, annealing at 55 °C for 90 s and extension at 72 °C for 2 min. The thermal profile ended with a final extension at 72 °C for 7 min. All PCR amplification products were analyzed by agarose gel (1.5%) electrophoresis [26].

### 2.6. Light Cycler PCR (LC-PCR)

Real-time PCR reactions were carried out with a LightCycler instrument using the QuantiTect™ SYBR^®^ Green PCR Kit (Qiagen, Hilden, Germany) with 0.3 μM of each primer, 5 mM MgCl_2_ and 5 μL of DNA template. DNA was replaced by sterile water in the negative control. The program used for real time PCR was 10 min at 95 °C, followed by 35 cycles of 15 s denaturation at 95 °C, 30 s annealing at 60 °C, 30 s elongation at 72 °C. Samples were placed into a glass capillary, capped, centrifuged for a few seconds in a micro-centrifuge using appropriate adapters, and then placed into the LightCycler rotor. In addition, the PCR products were recovered from the capillaries and analyzed by agarose gel (1.5%) electrophoresis and stained with ethidium bromide [27].

### 2.7. Specificities and Sensitivities of PCR Amplifications

The specificities of the gene-specific primers designed in this study were tested in PCR amplifications using purified genomic DNA from various fungal species as template. To assess the sensitivity of the detection of *M. graminicola* using the designed primers, DNA dilution series containing 10 ng to 50 fg of DNA from selected isolates were subjected to conventional, and real-time PCR analyses, respectively.

## 3. Results

### 3.1. Specificity and Sensitivity of the PCR Assays

**Figure 1:**
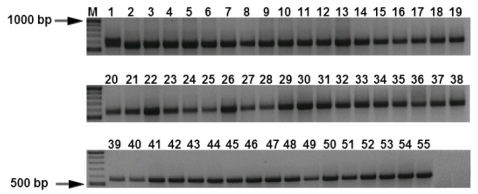
Polymerase chain reaction (PCR) amplification of genomic DNA isolated from 55 isolates of *M. graminicola* using primer pair (CT) C9 showing a amplification product of 570 bp. Lane M, 100 bp DNA marker; arrows indicate the 1000 and 500 bp DNA marker.

**Figure 2:**
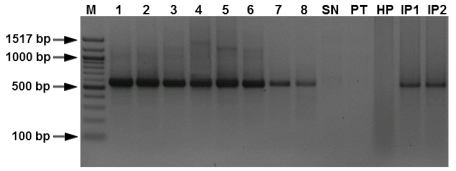
Polymerase chain reaction (PCR) amplification performed to assess specificity of the primer pair (CT) C9. A PCR fragment of 570 bp could only be amplified from Lanes 1–8 of genomic DNA from different isolates of *Mycosphaerella graminicola*, lane 9, DNA from *Stagonospora nodorum* (SN), lane 10, DNA from *Pseudocercosporella herpotrichoides* (PT), lane 11, healthy plant (HP), and lane 12–13, plants infected by *M. graminicola* (IP1 and IP2). Lane M, 100 bp DNA marker.

**Figure 3:**
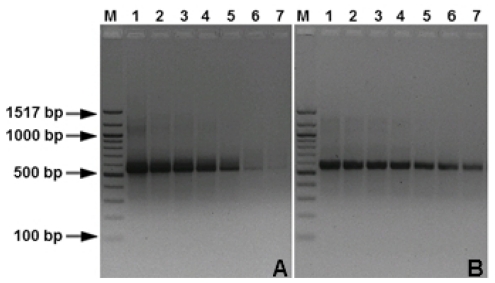
Comparison of the sensitivity of conventional (**A**) and real-time PCR (**B**), using different concentrations of *Mycosphaerella graminicola* DNA. For both (A) and (B), Lane 1, 10 ng; lane 2, 1 ng; lane 3, 100 pg; lane 4, 10 pg; lane 5,1 pg; lane 6, 100 fg; lane 7, 50 fg. Lane M, 100 bp DNA marker.

The specificity of the real-time PCR assay was tested with template DNA extracted from the 55 isolates of *M. graminicola* and other fungal species that are listed in Table 1. Dinucleotide specific-primer amplified a single fragment from total genomic DNA of *M. graminicola* (Figure 1) and its inoculated leaves. There was no amplification obtained in healthy plants (HP) or the other fungal pathogens tested (*Stagonospora nodorum* (SN), *Pseudocercosporella herpotrichoides* (PT)) (Figure 2). The (CT) 7 G primer amplified a unique DNA fragment of approximately 570 bp from *M. graminicola*, whereas no amplification was achieved with DNA isolated from other fungal species. Using DNA extracted from *M. graminicola* cultures, the detection limit was 100 pg/μL with conventional PCR, whereas the detection limit was 50 fg/μL by real-time PCR, thus the real-time PCR assay was 20-fold more sensitive than conventional PCR (Figure 3).

### 3.2. Melt Curve Analysis

**Figure 4:**
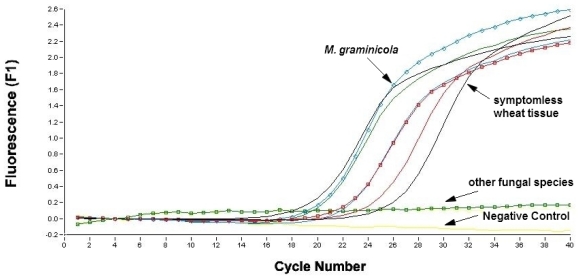
Amplification plot of fungal DNA from *Mycosphaerella graminicola* and other fungal species, as well as in symptomless wheat tissue and a non-template negative control by using a LightCycler instrument and (CT) C9 primer pair.

In the assay, SYBR Green I was used as the fluorescent dye enabling real-time detection of PCR products. Characterization of the amplicons was achieved by melting point analysis 84 ± 0.2 °C. Nonspecific products such as primer dimers could readily be distinguished from PCR products by their lower melting points. PCR reactions performed on 50 ng of *M. graminicola* DNA with primers targeted at fungal sequences led to similar *C*t and dissociation curves compared to a control devoid of DNA matrix, showing the specificity of fungal quantification (Figure 2). The system enables a 35-cycle PCR with 35 samples to be completed in 45 min, including quantification and identification of the product. Typical results from the probe melt curve analysis are shown in Figure 4. All products were subjected to analytical gel electrophoresis to confirm the 570 bp amplicon size.

### 3.3. Quantification of *M. graminicola* in Infected Plants

**Figure 5:**
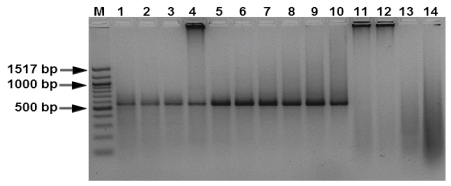
Polymerase chain reaction amplification performed to diagnose *Mycosphaerella graminicola* in artificially inoculated wheat leaves by (CT) C9 primer pair (product 570 bp). Lanes 1 to 10, DNA from inoculated leaves sampled at 0, 2, 6, 8, 10, 12, 14, 16, 18 and 20 days post inoculation with *M. graminicola;* lanes 11–12, DNA from healthy plants; lane 13, DNA from leaves inoculated with *Pseudocercosporella herpotrichoides*; lane 14, leaves inoculated d with *Fusarium graminearum.* Lane M, 100 bp DNA marker.

Artificially *M. graminicola****-***inoculated asymptomatic wheat leaves were collected and fungal DNA was detected with conventional PCR and real-time PCR 0, 2, 6, 8, 10, 12, 14, 16, 18 and 20 days post inoculation. Inoculated wheat leaves showed the 750-bp fragment that is diagnostic for *M. graminicola* infections after PCR with dinucleotide specific primers. No PCR products were generated with DNA isolated from *Fusarium graminearum*-inoculated or *Pseudocercosporella herpotrichoides-*inoculated wheat plants or from DNA isolated from healthy wheat tissue. A clear single DNA fragment was amplified from the samples taken on day 0, 2, 6, 8, 10, 12, 14, 16, 18 and 20 after inoculation (Figure 5, lanes 1–10). From inoculated wheat leaves harvested at day 0, although thoroughly washed with water prior to PCR, a clear fragment was still present representing the inoculated fungus. The intensity of the amplified DNA fragment increased significantly at 10 days after inoculation. Between 10 and 20 days after inoculation, the intensity of the amplified fragment increased more than five-fold. As mentioned before, the dinucleotide-specific primers can detect the pathogen from 10 pg of DNA isolated from infected leaf tissue in conventional PCR assays and from 50 fg in real-time PCR assays; the higher sensitivity offered by the real-time PCR assay makes it more reliable for detecting the fungus during the latent stage of infection. The sensitivity of the method would allow the quantification of fungal growth in different plant tissues during the progress of infection.

## 4. Discussion

Microsatellites have been used as genetic markers in numerous DNA and PCR fingerprinting trials for strain typing of a variety of filamentous fungi and yeasts without prior knowledge of their abundance and distribution in the investigated fungal genomes [21,28,29]. Here, we describe the use of conventional and real-time PCR using fluorescent SYBR Green I dye to quantify *M. graminicola* present in wheat tissue.

The SYBR Green dye has no sequence specificity and therefore does not require the design of specific fragments complementary to the target DNA. It can be used to detect any double-stranded (ds) DNA PCR product [30]. In the current research, positive amplification was conducted under a melting temperature of 84 °C. Therefore, non-specific amplification and primer dimers could easily be distinguished from the authentic amplicon pool. The values of the cycle threshold (*C*t) linearly correlated with the concentration of the target DNA, indicating that the method is suitable for qualitative and quantitative assay.

Specific dinucleotide primers amplified a single PCR fragment of 570 bp specific for isolates of *M. graminicola*, but no fragment was generated from 14 other fungal species belonging to 13 different genera. The primers amplified similar-sized fragments from fungus culture grown *in vitro* and wheat leaves inoculated with *M. graminicola*. This primer pairs allowed a reliable DNA quantification at concentrations as low as 50 fg, which is very sensitive, as reported by other researchers [12,14,15]. Real-time PCR was found to be more sensitive than the standard amplification procedure, and a successful amplification was obtained with *M. graminicola* DNA. Dinucleotide and trinucleotide repeats have higher probabilities of polymorphism than mononucleotide repeats [31]. The visual diagnostics of disease is difficult due to the presence of different *Mycosphaerella* species within one field and even on one plant, and the difficulty in distinguishing symptoms caused by biotic and abiotic stress. Identification of *Mycosphaerella* species based on morphology and presence of fruiting bodies and conidia is difficult due to the considerable morphological similarity of this fungus with other closely related fungi [16].

Here we compared conventional PCR and real-time PCR assays, which enabled us to detect the pathogen on asymptomatic plants collected two days after artificial inoculation. It has also been shown that this assay can be used to monitor fungal development in wheat tissue during the course of infection, even the starting inoculum could be detected immediately after washing of the inoculated leaves.

Fraaije *et al.* [12] did not detect an exponential increase in biomass until 14 days post inoculation using the PCR/PicoGreen assay. In our case using SYBR Green, we observed a significant increase at 10 days after inoculation. Real-time PCR was more sensitive than conventional PCR and can be used for routine quantitative analysis of *M. graminicola* in wheat tissue to trace new infections. This will be especially important when the target DNA concentration is low or PCR inhibitory substances are present [32].

Biomass accumulation of avirulent isolates of *M. graminicola* on resistant hosts could also be assessed by TaqMan® quantitative PCR using mating-type-specific probe and primer combinations, and results showed that the biomass of avirulent isolates on resistant host species and cultivars was either maintained or increased over time [33].

Accurate, rapid, and early detection of *M. graminicola* on wheat will assist the development of sustainable disease control and management.

## Figures and Tables

**Table 1:** List of *Mycosphaerella graminicola* isolates and isolates of other fungal species used to evaluate the specificity of the primers developed for identification and detection of *M. graminicola.*

Isolate Code	Fungal species	Host	Origin	PCR specificity^a^
K-Or-1	*M. graminicola*	Wheat	Germany	•
K-Or-30	*M. graminicola*	Wheat	Germany	•
K-Or-38	*M. graminicola*	Wheat	Germany	•
K-Or-44	*M. graminicola*	Wheat	Germany	•
OK-102	*M. graminicola*	Wheat	Germany	•
OK-108	*M. graminicola*	Wheat	Germany	•
OK-109	*M. graminicola*	Wheat	Germany	•
OK-112	*M. graminicola*	Wheat	Germany	•
OK-120	*M. graminicola*	Wheat	Germany	•
K-Ba-10	*M. graminicola*	Wheat	Germany	•
K-Ba-20	*M. graminicola*	Wheat	Germany	•
K-Ba-30	*M. graminicola*	Wheat	Germany	•
K-Ba-40	*M. graminicola*	Wheat	Germany	•
K-Ba-60	*M. graminicola*	Wheat	Germany	•
G-Or-1	*M. graminicola*	Wheat	Germany	•
G-Or-6	*M. graminicola*	Wheat	Germany	•
G-Or-8	*M. graminicola*	Wheat	Germany	•
G-Or-88	*M. graminicola*	Wheat	Germany	•
G-or-98	*M. graminicola*	Wheat	Germany	•
G-Or-102	*M. graminicola*	Wheat	Germany	•
M-or-1	*M. graminicola*	Wheat	Germany	•
M-or-4	*M. graminicola*	Wheat	Germany	•
M-Or-8	*M. graminicola*	Wheat	Germany	•
M-Or-82	*M. graminicola*	Wheat	Germany	•
M-Or-98	*M. graminicola*	Wheat	Germany	•
M-or-102	*M. graminicola*	Wheat	Germany	•
L-Or-1	*M. graminicola*	Wheat	Germany	•
L-Or-8	*M. graminicola*	Wheat	Germany	•
L-Or-84	*M. graminicola*	Wheat	Germany	•
L-Ba-1	*M. graminicola*	Wheat	Germany	•
L-Ba-8	*M. graminicola*	Wheat	Germany	•
L-Ba-110	*M. graminicola*	Wheat	Germany	•
H-Or-1	*M. graminicola*	Wheat	Germany	•
H-Or-8	*M. graminicola*	Wheat	Germany	•
H-Or-90	*M. graminicola*	Wheat	Germany	•
H-Ba-103	*M. graminicola*	Wheat	Germany	•
H-Ba-104	*M. graminicola*	Wheat	Germany	•
H-Ba-116	*M. graminicola*	Wheat	Germany	•
CH1	*M. graminicola*	Wheat	Switzerland	•
CH2	*M. graminicola*	Wheat	Switzerland	•
CH3	*M. graminicola*	Wheat	Switzerland	•
CH4	*M. graminicola*	Wheat	Switzerland	•
CH5	*M. graminicola*	Wheat	Switzerland	•
FCH1	*M. graminicola*	Wheat	France	•
FCH2	*M. graminicola*	Wheat	France	•
FC1	*M. graminicola*	Wheat	France	•
FC2	*M. graminicola*	Wheat	France	•
FN4	*M. graminicola*	Wheat	France	•
FN5	*M. graminicola*	Wheat	France	•
GBW1	*M. graminicola*	Wheat	England	•
GBW2	*M. graminicola*	Wheat	England	•
GBE2	*M. graminicola*	Wheat	England	•
GBE4	*M. graminicola*	Wheat	England	•
GBN1	*M. graminicola*	Wheat	England	•
GBN2	*M. graminicola*	Wheat	England	•
FOV	*Fusarium oxysporum* f. sp. *vasinfectum*	Cotton	Egypt	○
FS	*Fusarium solani*	Cotton	Egypt	○
FG	*Fusarium germanium*	Wheat	Germany	○
FP	*Fusarium poae*	Wheat	Germany	○
MP	*Macrophomina phaseolina*	Cotton	Egypt	○
SP	*Septoria passerinii*	Barely	USA	○
TH	*Trichoderma harzianum*	Cotton	Egypt	○
SN	*Stagonospora nodorum*	Wheat	Germany	○
PT	*Pyernophora teres*	Wheat	Germany	○
PTR	*Pyernophora tritici-repentis*	Wheat	Germany	○
PH	*Pseduosercosporella heropotrichoides*	Wheat	Germany	○
Pen	*Pencillium* sp.	Unknown	Egypt	○
Alt	*Alternaria* sp.	Unknown	Egypt	○
CB	Ce*rcospora beticola*	Suger beet	Germany	○

a The presence or absence of species-specific amplicon is indicated by a positive (●) or negative (○) for each set of primers.
